# Enhancing Evidence Synthesis Efficiency: Leveraging Large Language Models and Agentic Workflows for Optimized Literature Screening

**DOI:** 10.1002/cesm.70042

**Published:** 2025-10-21

**Authors:** Bing Hu, Emmalie Tomini, Tricia Corrin, Kusala Pussegoda, Elias Sandner, Andre Henriques, Alice Simniceanu, Luca Fontana, Andreas Wagner, Stephanie Brazeau, Lisa Waddell

**Affiliations:** ^1^ Data Management, Innovation and Analytics, Data, Surveillance and Foresight, Public Health Agency of Canada Ottawa Ontario Canada; ^2^ Health Canada Ottawa Ontario Canada; ^3^ University of Waterloo Waterloo Ontario Canada; ^4^ National Microbiology Laboratory Public Health Risk Sciences, Public Health Agency of Canada Ottawa Ontario Canada; ^5^ Technical University of Graz Graz Austria; ^6^ CERN (European Organization for Nuclear Research) Graz Austria; ^7^ Health Emergencies Programme WHE, World Health Organization Geneva Switzerland

## Abstract

**Background:**

Public health events of international concern highlight the need for up‐to‐date evidence curated using sustainable processes that are accessible. In development of the Global Repository of Epidemiological Parameters (grEPI) we explore the performance of an agentic‐AI assisted pipeline (GREP‐Agent) for screening evidence which capitalizes on recent advancements in large language models (LLMs).

**Methods:**

In this study, the performance of the GREP‐Agent was evaluated on a data set of 2000 citations from a systematic review on measles using four LLMs (GPT4o, GPT4o‐mini, Llama3.1, and Phi4). The GREP‐Agent framework integrates multiple LLMs and human feedback to fine‐tune its performance, optimize workload reduction and accuracy in screening research articles. The impact on performance of each part of this Agentic‐AI system is presented and measured by accuracy, precision, recall, and F1‐score metrics.

**Results:**

The results show how each phase of the GREP‐Agent system incrementally improves accuracy regardless of the LLM. We found that GREP‐Agent was able to increase sensitivity across a broad range of open source and proprietary LLMs to 84.2%–88.9% after fine‐tuning and to 86.4%–95.3% by varying workload reduction strategies. Performance was significantly impacted by the clarity of the screening questions and setting thresholds for optimized workload reduction strategies.

**Conclusions:**

The GREP‐Agent shows promise in improving the efficiency and effectiveness of evidence synthesis in dynamic public health contexts. Further development and refinement of adaptable human‐in‐the‐loop AI systems for screening literature are essential to support future public health response activities, while maintaining a human‐centric approach.

## 1 Introduction

Recent public health emergencies of international concern have underscored the importance of enhancing public health organizations’ capabilities to acquire and analyze epidemiological data to support modelling, risk assessment, guideline development and other decision making. The creation of the Global Repository of Epidemiological Parameters (grEPI), an accessible living repository of epidemiological parameters for priority infectious pathogens, is the vision of the EpiParameter community [1]. GrEPI will be publicly accessible by modelers, epidemiologists, subject matter experts and decision makers to inform mathematical models and support public health response activities. This will strengthen global public health and prevent redundant efforts in synthesizing epidemiological evidence [2, 3, 4].

The global need to have epidemiological parameters on priority infectious diseases maintained in an up‐to‐date repository has been evident during event response activities in the last decade [1, 2, 4]. Capitalizing on epidemiological parameter systematic reviews recently undertaken by members of the EpiParameter community [5, 6, 7, 8], an artificial intelligence (AI) assisted pipeline is being built to automate the identification and extraction of epidemiological parameters of priority infectious diseases from scientific literature using living systematic review methodology [1, 9]. The integration of AI will help achieve a sustainable living repository that is underpinned by a structured, reproducible, and auditable process for adding evidence.

The recent evolution of generative AI, particularly large language models (LLMs), suggests that LLMs could play a pivotal role in automating many steps of an evidence synthesis process, reducing workload and reserving expert time for analysis [10, 11, 12]. Many studies have evaluated different LLMs’ ability to evaluate and classify articles providing accuracy and recall data relative to curated data based on the current standard of two human reviewer screening [10, 13, 14, 15, 16, 17]. There are several considerations in the implementation of LLMs for screening within the evidence synthesis process that are not mutually exclusive and should be considered as components of a system each with a role to play in optimizing performance [18]. These include how AI can be part of a system to screen articles that is easily audited to verify performance, allows performance thresholds to be set, and can determine when human input is needed based on the workload reduction strategies in place [12]. In this case, an agentic AI approach is employed to capitalize on agents operating independently to screen citations, Appendix A. The versatility of the system is also important as generative AI is still evolving as are the policies and practices around their use, thus the LLMs used in the system should be interchangeable. User skillsets need to be developed to efficiently translate a systematic review protocol into optimized prompts for LLM screening.

The objective of this project is to evaluate the use of LLMs in screening and classification of research within an agentic AI screening workflow, referred to as the GREP‐Agent. Through optimizing the performance of LLMs in screening and classification of research, we demonstrate how this can work in real‐time and estimate the human workload reduction potential compared to dual screening by human reviewers.

## 2 Methodology

The following describes the development and testing of the GREP‐Agent system which has been designed to focus on a human‐centric approach for LLM title and abstract literature screening. In this model the human reviewer plays a critical role in the workflow providing feedback to the LLM to calibrate the GREP‐Agent screening system. To evaluate the performance of the GREP‐Agent, we utilize a data set of 2000 double screened citations from an ongoing systematic review extracting epidemiological parameters for measles [19]. Additional details on the measles data set and screening questions can be found in Appendix B. In the following section we cover the core architecture for GREP‐Agent as well as the GREP‐Agent framework.

**Figure 1:**
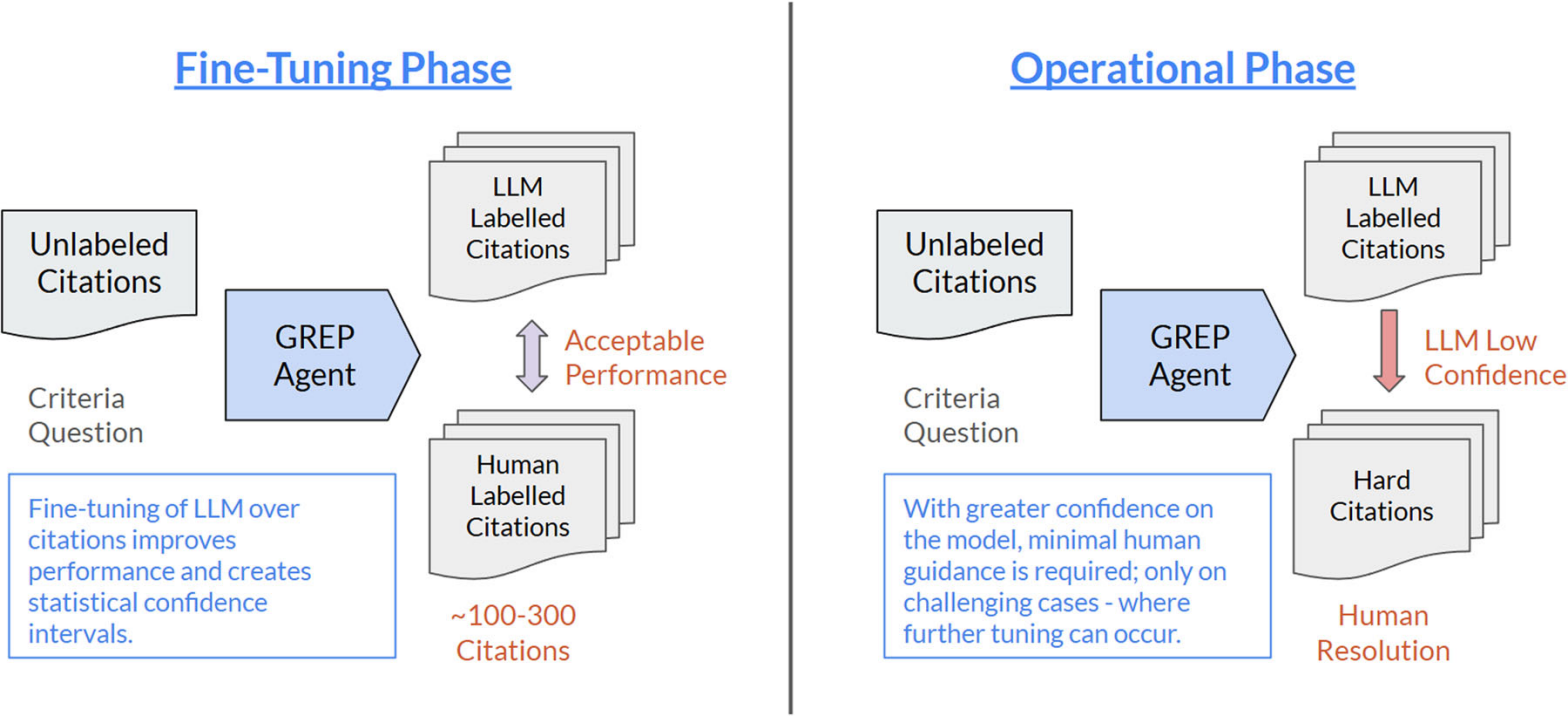
The two phases of GREP‐Agent; [1] the fine‐tuning phase, and [2] the operational phase.

The GREP‐Agent framework, as shown in Figure 1, consists of two phases: [1] the fine‐tuning phase, and [2] the operational phase. In the fine‐tuning phase the model starts with prompts analogous to systematic review screening criteria, human reviewers then optimize the model performance over a small subset of 100 to 300 LLM labelled citations by providing feedback to the GREP‐Agent on citations where the human disagrees with the LLM. Human reviewers can analyze these disagreements to further refine and edit criteria prompts used by the GREP‐Agent to improve screening performance, this iterative phase may include testing different versions of the question or context provided to the LLM. Suitable thresholds and parameters for workload reduction strategies are selected during fine‐tuning usually with the objective to balance achieving high sensitivity with maximum workload reduction, see results and Appendix C for additional details.

When an acceptable performance level, as defined by scientific evaluators, is reached, the operational phase begins. The operational phase applies the fine‐tuned prompts for GREP‐Agent to the remaining unlabeled citations. Strategies to realize workload reduction are applied including processing citations based on multi‐agent agreement and LLM produced confidence levels, explained in Section 3.2.2. Using both strategies, our results show how challenging citations with low LLM confidence levels and multi‐agent disagreement are presented to human reviewers for additional feedback and labelling. The human workload is reduced for citations where the model has high confidence and multi‐agent agreement.

### 2.1 GREP‐Agent Architecture

**Figure 2:**
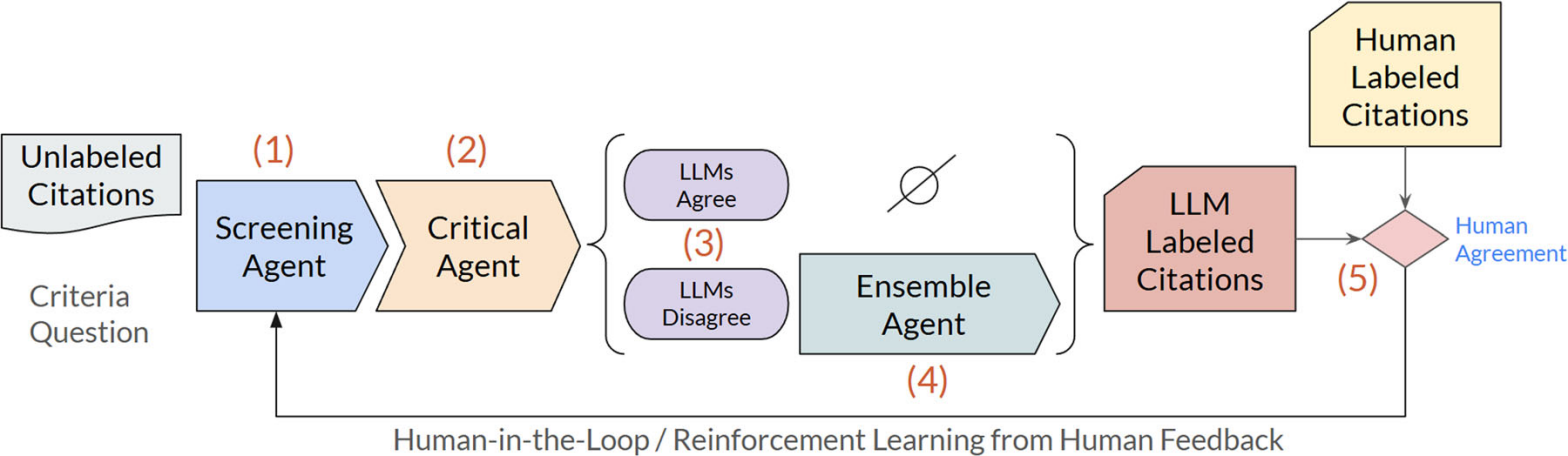
The GREP‐Agent architecture. Three LLM agents make up the core of our architecture; [1] the screening agent, [2] the critical agent, and [3] the ensemble agent.

Figure 2 shows the GREP‐Agent architecture which consists of 5 steps: (1) the screening agent, which completes the first screening of the citation; (2) the critical agent, which re‐evaluates the first screening of the citation; (3) logic that determines if the screening and critical agents agree or disagree; (4) the ensemble agent, which consists of multiple additional randomized screening agents completing screening based on majority vote; and (5) the human‐in‐the‐loop reinforcement learning, where human feedback is used to fine‐tune the system. In this agentic AI approach each agent runs autonomously, with the output of the previous agent in the workflow changing the behavior of following agents, such as the critical agent challenging the answer selected by the screening agent, and the ensemble agent only running if there is a disagreement between the screening agent and the critical agent.

The use of agentic LLMs for the GREP‐Agent enables a human‐centric AI approach for literature screening. Reviewers have granular control over the behavior of each agent, allowing for the incorporation of feedback, optimizing the performance for individual inclusion/exclusion criteria. By tracking the behavior of each agent, through reported confidence levels, and multi‐agent agreements or disagreements, workload reduction can be realized by only showing human reviewers challenging citations with lower LLM confidence or disagreement between multiple LLM agents. Human feedback provided on the challenging citations is used by the GREP‐Agent to further refine the behavior of the agents for improved performance. Models and hyperparameters can be specified for each agent. System prompts, models, and hyperparameters used in this study are included in Appendix D.

#### 2.1.1 The Screening Agent

**Figure 3:**
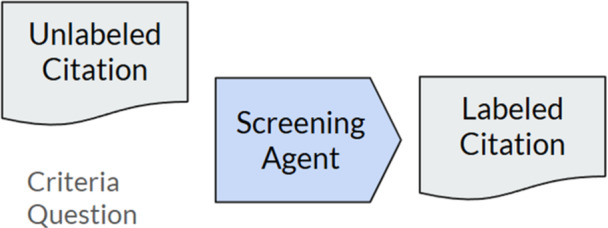
The screening agent produces a labeled citation through evaluating the citation using the provided criteria.

The screening agent, as shown in Figure 3, is a LLM that takes as input a system prompt (more in Appendix B and D) containing an unlabeled citation and a criteria question to provide a structured output of the LLM label of the citation based on the criteria, the confidence level between 0 and 1 generated by the LLM in the provided answer, and a brief explanation of the reasoning of the LLM.

#### 2.1.2 The Critical Agent

**Figure 4:**
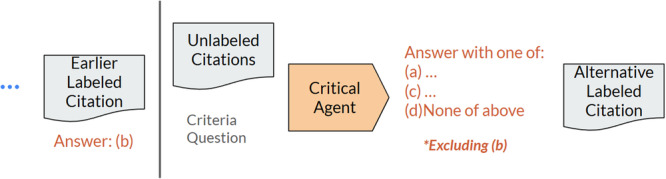
The critical agent. Given the earlier answer from the screening agent, the critical agent must select a new label for the citation from a modified list of choices, excluding the previous screening agent answer, and including an additional option of “None of Above.” The critical agent provides an alternative answer that may deviate from the original agent.

The critical agent, depicted in Figure 4, is designed to provide an alternative answer that may or may not deviate from the first screening agent. Deviation between the answers from the screening and critical agent may mean that it is challenging to apply and evaluate the citation using the provided criteria. The critical agent takes as input a modified system prompt, similar to the screening agent, containing the same citation and criteria but with a modified list of possible criteria labels to select from (more in Appendix D). The modified list of criteria labels is the original criteria but with the removal of the screening agent answer and the addition of a “None of Above” option. The output of the critical agent includes one of the labels in the modified list of criteria labels, a confidence score, and a brief explanation of the reasoning of the LLM.

Agreement between the screening and critical agent is determined logically by comparing the two answers. In the case where the critical agent answers with “None of Above,” we count this as an agreement between the critical and screening agents. In the other case where the critical agent answers with one of the other options, there is disagreement between the screening and critical agent. In cases of agreement, the answer provided by both the screening agent and the critical agent becomes the final LLM label. Records with disagreement between the screening and critical agents undergo an additional step, called the ensemble agent, for further evaluation.

#### 2.1.3 Ensemble Agent

**Figure 5:**
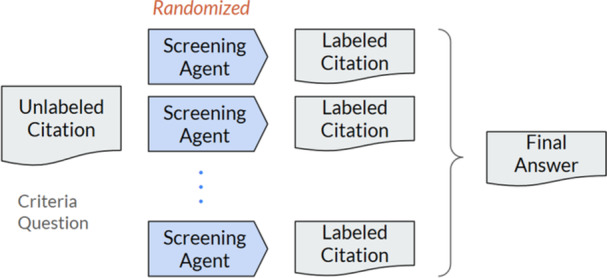
The ensemble agent.

The ensemble agent, as shown in Figure 5, is a collection of screening agents each run with randomized parameters of model, temperature, seed, and top‐p (Appendix C). Given the differences in parameters, the screening agents may give answers that deviate from each other. The majority vote of all ensemble and original screening agents determines the final label for the citation. The ensemble agent is designed to only run when there is uncertainty and disagreement. This approach reduces the cost of LLMs by at least 33% compared to running the ensemble agent on all citations to produce consensus votes.

#### 2.1.4 Reinforcement Learning from Human Feedback

**Figure 6:**
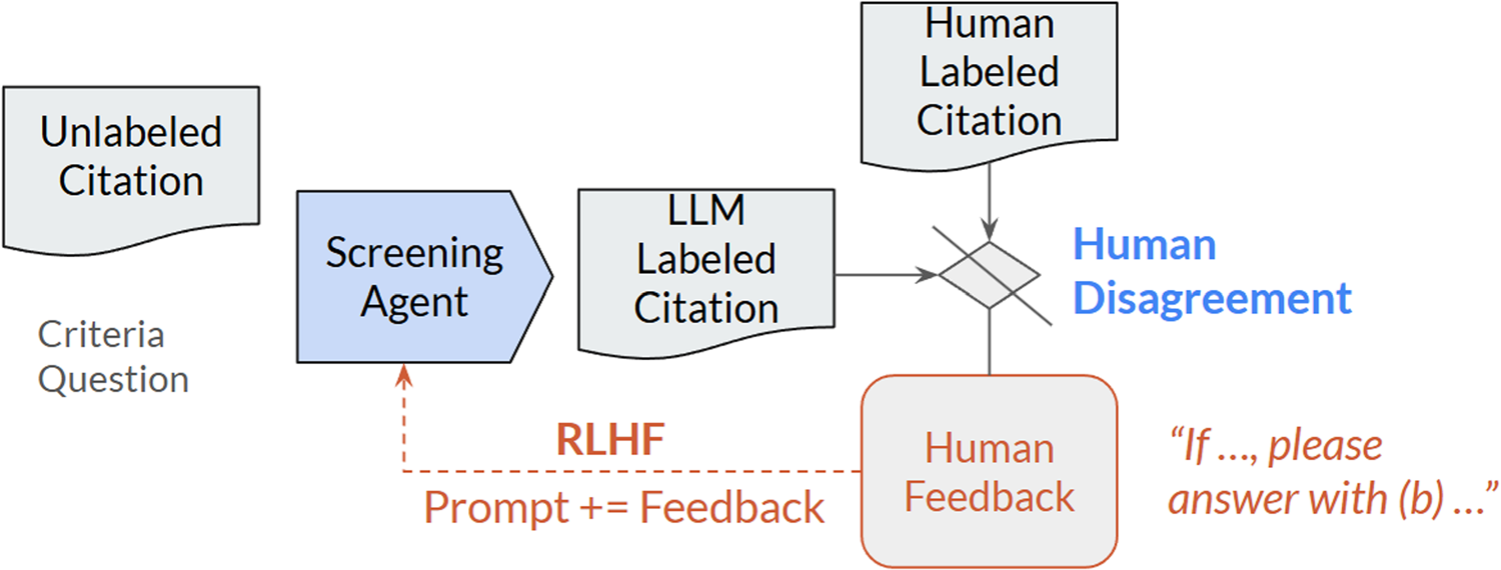
The reinforcement learning from human feedback (RLHF) process. When there is disagreement between the LLM and human reviewers for a citation, feedback from humans is collected to further improve and refine the criteria prompt to fine‐tune and improve GREP‐Agent performance.

Figure 6 shows how reinforcement learning from human feedback improves the performance of the GREP‐Agent. When there is a disagreement between the LLM and human labels for a citation, the human reviewer can analyze the reasoning provided by the LLM and provide feedback to the screening agent to clarify or add criteria for the LLM to use. Human feedback is directly incorporated into the prompt provided to the agents to help the LLM contextualize nuance and implicit criteria that the LLM was missing or did not understand. Reviewers can input feedback whenever there is a LLM and human disagreement, at fine‐tuning the feedback is appended to the prompt and used by the LLM when screening. This fine‐tuning aims to adjust the LLM towards the desired behavior, leading to improved overall agent performance and detection of challenging citations. During the operational phase human feedback may also be provided to the LLM on citations that the LLM was not confident on and pushed to human reviewers.

## 3 Results

This project used 2000 citations that were dual screened in a systematic review. The 2000 citations had the following characteristics, 37.7% were included in the review as they met all three screening criteria: 95.8% were on the human population; 45.8% were on measles disease; and 75.1% were primary research, 23% were non‐primary research, 1.1% were conference abstracts, and 0.8% were evidence syntheses. This represents a good variety in the sample citations, while balancing the cost and time required to conduct this evaluation. The performance of the GREP‐Agent framework with four different LLMs (GPT4o, GPT4o‐mini, Llama3.1, and Phi4) was measured by accuracy, precision, recall (i.e., sensitivity), and F1‐score metrics.

### 3.1 Retrospective Study

Table 1 contains retrospective evaluation metrics based on the measles data set of the GREP‐Agent using different LLMs after fine‐tuning on the three screening questions: [1] is this article primary research, [2] is this article on the human population, and [3] is the main focus of this study about measles disease. The overall screening result is the combination of answers from these three screening questions (Appendix B). These results reflect the baseline performance of applying the GREP‐Agent without human intervention at 100% workload reduction.

**Table 1:** Performance of GREP‐Agent models after fine‐tuning on 2k measles data set.

Criteria	Model	Acc (%)	F1 (%)	Precision (%)	Recall (%)
Study type question	GPT4o	**93.8**	**93.9**	94.5	**94.0**
	GPT4o‐mini	89.5	88.6	91.2	89.5
	Llama3.1*	84.4	83.3	**94.8**	84.4
	Phi4	91.1	90.8	91.8	91.1
Human population question	GPT4o	**97.8**	98.0	98.3	**97.8**
	GPT4o‐mini	97.8	**98.1**	**98.7**	97.8
	Llama3.1*	97.2	97.4	97.7	97.2
	Phi4	97.7	98.0	98.7	97.7
Measles question	GPT4o	87.4	87.6	**89.6**	87.4
	GPT4o‐mini	89.1	89.1	89.1	89.1
	Llama3.1*	85.7	85.6	85.7	85.7
	Phi4	**89.3**	**89.3**	89.3	**89.3**
**Overall**	GPT4o	88.1	88.4	**89.4**	88.1
	GPT4o‐mini	88.2	88.2	88.2	88.2
	Llama3.1*	84.2	84.2	84.3	84.2
	Phi4	**88.9**	**88.8**	88.8	**88.9**

* Llama3 8b parameter. The best performances for each question criteria and metric are bold.

### 3.2 GREP‐Agent Workflow

GREP‐Agent applies several strategies to reduce human workload while maximizing consistent performance of LLM screening across the screening criteria and citations. By using human verification for citations that the LLM finds most challenging, GREP‐Agent can maximize sensitivity while minimizing the human workload through setting confidence thresholds and critical agent parameters.

#### 3.2.1 Prompt Engineering and Finetuning Phase

In this section we show how developing good screening criteria prompts and fine‐tuning improves performance of the GREP‐Agent compared to the human labelled data set (the control data). Specific wording used in the screening criteria prompt can have a large effect on the overall performance.

In Table 2 we see how simply changing the wording of the question without changing any of the context provided to the LLM can alter the model's performance. This suggests that designing clear questions with minimal subjectivity or nuance directly impacts LLM performance and developing prompt engineering skills is critical to using LLMs for literature screening.

**Table 2:** Effects of changing the screening criteria measles question while keeping the overall criteria constant.

Measles ablation	Specific wording	Acc (%)	F1 (%)	Precision (%)	Recall (%)
Original question	Is the main focus of this study about measles disease?	87.4	87.6	89.6	87.4
Updated question	*Is this study reporting on measles disease?*	81.1	81.0	81.6	81.1

*Note:* Results gathered for GREP‐Agent GPT4o.

**Figure 7:**
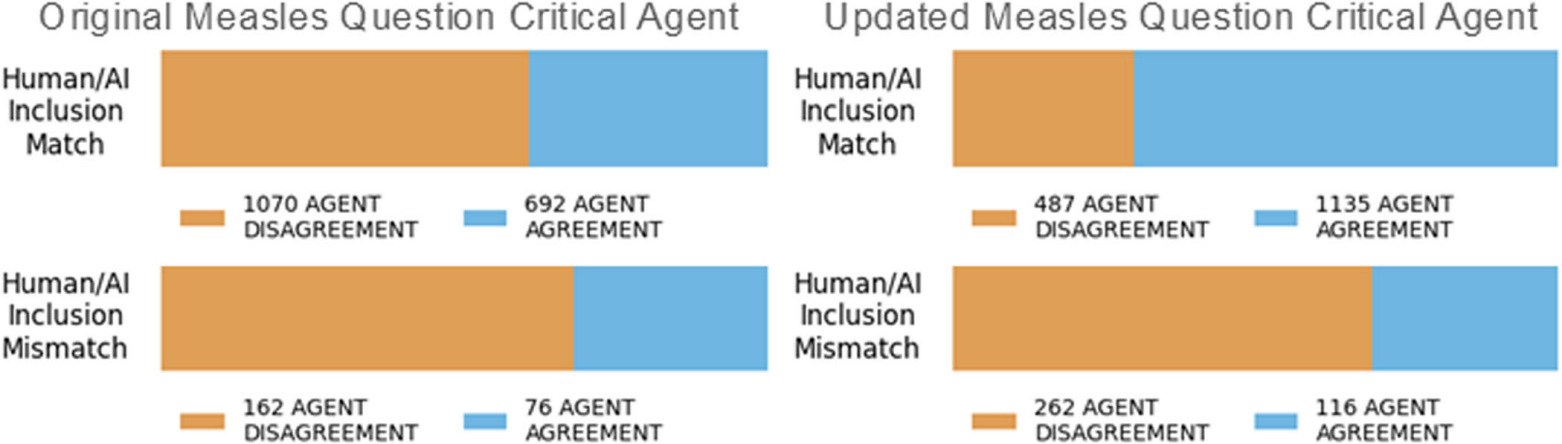
Comparing GPT4o critical agent confusion matrix between the original and updated measles questions for human/AI match or mismatch. Agent disagreement is when the screening and critical agent responses do not match and vice versa for agent agreement.

In this example we found that the usage of “main focus” in the original *question* had higher performance statistics, but in finetuning it was noted that it also suffered from confusion about inclusion and exclusion that could not be resolved. This introduced confusion primarily impacts sensitivity of our workload reduction strategies to single out challenging citations for human verification. On the other hand, although the more general question has worse statistics at the fine‐tuning phase, there was better sensitivity when combined with the workload reduction strategies because more citations were flagged for human review (Figure 7) leading to better overall performance during the operational phase. In this example it was equally important to consider the sensitivity of the chosen workload reduction strategy as well.

Table 3 shows the effectiveness of screening criteria prompt fine‐tuning for a GREP‐Agent, which is achieved by a human reviewer evaluating and correcting the LLM on 100–300 citations. The original and fine‐tuned screening criteria prompts can be found in Appendix B.

**Table 3:** Results of screening criteria fine‐tuning on a GREP‐Agent GPT4o‐mini model.

Criteria	Fine‐tuning	Acc (%)	F1 (%)	Precision (%)	Recall (%)
Study type question	Before	82.5	80.1	85.9	82.5
	After	**89.5**	**88.6**	**91.2**	**89.5**
Human population question	Before	93.8	93.9	94.5	94.0
	After	**97.8**	**98.1**	**98.7**	**97.8**
Measles question	Before	88.9	89.0	89.0	88.9
	After	**89.1**	**89.1**	**89.1**	**89.1**
**Overall**	Before	85.9	85.8	85.8	85.9
	After	**88.2**	**88.2**	**88.2**	**88.2**
	Improvement	**2.3**	**2.4**	**2.4**	**2.3**

#### 3.2.2 Workload Reduction Strategies

**Figure 8:**
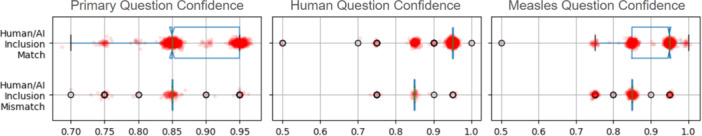
GREP‐Agent GPT4o reported confidence levels grouped by human/AI Inclusions match and mismatch.

During the operational phase, the GREP‐Agent utilizes strategies to isolate challenging citations that require human review and verification from those that do not. Citations that require human verification are those that are likely to have human/AI inclusion mismatch (Figure 8). Citations that fall outside of being challenging, those likely to have human/AI inclusion or exclusion match, can confidently be screened by the GREP‐Agent to effectively reduce the human reviewers’ workload. Two strategies that the GREP‐Agent uses to isolate these challenging citations are filtering by confidence from the screening agent and by agreement between the screening agent and the critical agent response.

Figure 8 shows the distribution of the screening agent's confidence levels compared to the human screened control data set grouped by human/AI match and mismatch. We see the model had a higher confidence level when there was a human/AI inclusion match. The Point Biserial Correlation Coefficients between inclusion match and mismatch and LLM reported confidences are 0.17, 0.30, and 0.42 for each study type, human population, and measles question respectively, which were statistically significant. In this example a screening agent confidence threshold for workload reduction of 0.9 bisects citations likely to have human/AI inclusion match from the remaining possibly challenging citations.

**Figure 9:**
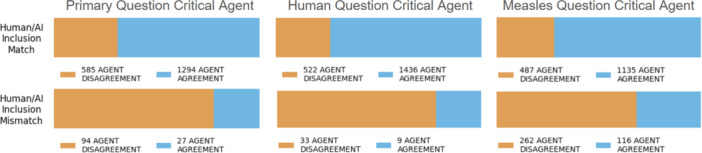
GREP‐Agent GPT4o critical agent likelihood between human/AI Inclusion match or mismatch. Agent disagreement is when the screening and critical agent responses do not match and vice versa for agent agreement.

The likelihood of critical agent disagreement, when the critical agent disagrees with the screening agent, between human/AI inclusion match or mismatch using the human screened control data set, is shown in Figure 9. From the human screened control data set, we see that the model is more likely to have critical agent disagreement for citations where there is a human/AI inclusion mismatch. The Matthews Correlation Coefficient (MCC) between inclusion match and mismatch and critical agent agreement and disagreement are −0.23, −0.16, and −0.31 for each study type, human population, and measles question, respectively, ranging between weak (0 to −0.3) and moderate (−0.3 to –0.7) negative correlations. Although the correlation is weak to moderate, it is sufficient to be combined with other workload reduction strategies effectively such as confidence thresholding. The MCC between the overall inclusion match and mismatch with overall critical agent agreement and disagreement is −0.14. Selecting citations for human review where there is critical agent disagreement can be an effective strategy for workload reduction.

#### 3.2.3 Operational Phase

The GREP‐Agent operational phase combines both workload reduction techniques of confidence and critical agent agreement filtering to effectively isolate citations that require human review from those that do not. Results are calculated with the assumption that citations pushed to human reviewers will be answered correctly. The results of applying both workload reduction strategies in the GREP‐Agent workflow are shown in Table 4. The GREP‐Agent using GPT4o isolated ~20% of citations for human review. Assuming the human review is accurate, the optimized performance of the GREP‐Agent was a workload reduction of 80% and an 11% improvement in performance.

**Table 4:** Performance of GREP‐Agent GPT4o with and without optimized performance in the operational phase by balancing human review and workload reduction.

Criteria	Human review	Workload reduction (%)	Acc (%)	F1 (%)	Precision (%)	Recall (%)
Study type question	None	100	93.8	93.9	94.5	94.0
	With	73.2	**98.1**	**98.1**	**98.1**	**98.1**
Human population question	None	100	97.8	98.0	98.3	97.8
	With	92.7	**99.4**	**99.4**	**99.5**	**99.4**
Updated measles question	None	100	81.1	81.0	81.6	81.1
	With	72.7	**93.7**	**93.7**	**94.2**	**93.7**
**Overall**	None	100	83.6	83.4	83.7	83.6
	With	79.6	**94.6**	**94.6**	**94.9**	**94.6**
	Improvement	—	**11.0**	**11.2**	**11.2**	**11.0**
Original measles question	None	100	87.4	87.6	89.6	87.4
	With	83.2	**90.1**	**90.3**	**92.2**	**90.1**
**Overall**	None	100	88.1	88.4	89.4	88.1
	With	86.2	**91.1**	**91.3**	**92.6**	**91.1**
	Improvement	—	**3.0**	**2.9**	**3.2**	**3.0**

*Note:* Both the original and updated measles questions, corresponding overall results, and improvements are included.

Table 5 summarizes the optimized performance of the GREP‐Agent across LLMs. There was performance consistency across LLMs (e.g., accuracy range: 86.4%–95.3%) with similar workload reductions (range: 71.2%–92.3%).

**Table 5:** Overall scores of the optimized performance of the GREP‐Agent across LLMs during the operational phase by balancing human review and workload reduction.

Model overall scores	Human review	Workload reduction (%)	Acc (%)	F1 (%)	Precision (%)	Recall (%)
GPT4o (UQ)	None	100	83.6	83.4	83.7	83.6
	With	79.6	**94.6**	**94.6**	**94.9**	**94.6**
GPT4o (OQ)	None	100	88.1	88.4	89.4	88.1
	With	86.2	**91.1**	**91.3**	**92.6**	**91.1**
GPT4o‐Mini	None	100	88.2	88.2	88.2	88.2
	With	71.2	**95.3**	**95.3**	**95.4**	**95.3**
Llama3.1*	None	100	84.2	84.2	84.3	84.2
	With	73.3	**86.4**	**86.4**	**86.4**	**86.4**
Phi4	None	100	88.9	88.8	88.8	88.9
	With	92.3	**92.1**	**92.1**	**92.1**	**92.1**

* Llama3.1 8B parameter model. Both the original (OQ) and updated (UQ) measles questions and corresponding overall results and improvements are included.

Comparing the original and updated measles question from Tables 4 and 5, we see there is a tradeoff between workload reduction and accuracy. There are two key principles; [1] higher fine‐tuning accuracies result in greater workload reduction, and [2] workload reduction strategies can be used to improve sensitivity by lowering the threshold for what constitutes a challenging citation in the operational phase. You would want to optimize both higher fine‐tuning accuracy as well as workload reduction sensitivities. A tradeoff may occur as higher fine‐tuning accuracy is not always correlated with greater workload reduction sensitivity, as demonstrated by comparing the original and updated measles questions. As LLMs are responsive to reasoning, optimizing both workload reduction and accuracy is possible by iteratively applying human feedback on LLM errors during the fine‐tuning process.

## 4 Discussion

The integration of AI into evidence synthesis has been an area of research for several years, and many studies have evaluated different types of AI‐assisted literature screening approaches [11, 12, 20, 21, 22, 23]. Many approaches to date required large training datasets and did not incorporate human monitoring into the process, thus limiting their application into evidence synthesis workflows. This paper describes the development and initial results of an agentic AI system that is designed to overcome some of these limitations. By incorporating human prompt fine‐tuning and adjustable confidence thresholds, the balance between workload reduction and performance of the system to automate the screening of citations can be optimized and will help overcome many performance issues identified by recent reviews on LLM integration into the evidence synthesis process [11, 12]. By design, the GREP‐Agent would be incorporated into an evidence synthesis project for screening, the questions and prompts needed for the agent are analogous to developing screening questions and inclusion and exclusion criteria at the protocol stage. To maximize performance the human then interacts with the GREP‐Agent to provide additional guidance during the fine‐tuning stage for 100–300 citations. This stage is used to evaluate the clarity of the prompts and set thresholds for workload reduction to achieve optimal performance. Finally, the deferral to humans for articles that are not confidently classified by the GREP‐Agent at the operational phase is a valuable addition to AI‐assisted screening.

Two reviewer screening is a fundamental part of most evidence synthesis processes; however, research has also shown that an element of error remains with this approach and that humans may introduce bias into the process based on their experience and judgments [24]. The GREP‐Agent, like other AI‐assisted workflows, can partially address this issue through uniform application of prompts that have been fine‐tuned by a human reviewer on a small number of citations, but also addresses concerns raised by other authors related to monitoring, auditing and knowing when the LLM assessment is uncertain [11, 12]. The LLM reasoning and decisions are available for the human reviewer to audit at any point, but should be particularly scrutinized during fine‐tuning, and all instances where the LLM reasoning is diverging from the intended inclusion and exclusion criteria should be investigated. During the operational phase the workflow can be adjusted to maximize sensitivity or maximize workload reduction depending on the objectives of the evidence synthesis project.

When applying an AI‐assisted work flow it is important to be cognizant of how the LLM performance is affected by the prompt language. In this case, we evaluated the framing of the screening questions and supporting inclusion and exclusion criteria developed at the beginning of the systematic review. Our results suggest that extra care should be taken to have clearly articulated criteria that are not nuanced or ambiguous. We also suggest iteratively using the fine‐tuning phase to critique the clarity of the screening questions and potentially refine them to improve the LLM interpretation. This step could be considered analogous to pre‐testing a screening tool with reviewers in a systematic review or other evidence synthesis project.

Workload reduction and speed of conducting a review are key motivators for integrating AI into the evidence synthesis process. Factored into this should be the time to develop or adapt an AI assisted system and the cost of using LLMs in the system. The GREP‐Agent has been developed to be easily adapted to new topics resulting in minimal set‐up time to apply the GREP‐Agent in future reviews, which will maximize the workload reduction potential of using AI in the review process. The GREP‐Agent has also been designed to minimize the number of unnecessary model runs by only running extra models when there is uncertainty and disagreement. We estimate this approach reduces the cost of LLMs by at least 33% compared to running five LLMs on all the citations to produce consensus votes.

The GREP‐Agent described in this paper has been designed with adaptability in mind. Future proofing the workflow includes an interface that is independent from the LLMs used, meaning the GREP‐Agent can be directed to change which models are used and can integrate new LLMs as they evolve. In practice this agentic approach to AI screening is very well suited to addressing some of the challenges of living systematic reviews, conducting evidence surveillance, or addressing topics underpinned by a lot of research [9, 25, 26]. The collation of epidemiological parameters on priority pathogens is an example of the need to develop sustainable methodologies for conducting living evidence syntheses [1, 2, 4]. Investing time into the development of these AI‐assisted systems that are flexible to new topics and are ready to implement will speed up the review process and leave more time for critical appraisals and synthesis of the evidence.

In this study we used a curated data set to evaluate the performance of the GREP‐Agent, however one of the goals is to move away from large, curated datasets. For this we consider that when using an established AI tool there are some validation and verification steps built into the systematic review process that could be used to gauge how well a tool like the GREP‐Agent performed on a new review topic and if there are concerns with omission of relevant research. This includes using a list of seed articles that the review team knows are relevant to monitor the fate of those articles during screening with the GREP‐Agent. This list is usually curated when developing the review protocol and search strategy. Search verification strategies of checking reference lists of relevant articles or reviews for references omitted by the search strategy could also be used to check that relevant articles were included in the review by the GREP‐Agent. Extending these systematic review steps to GREP‐Agent screening evaluation allows some validation of performance and an opportunity to explore the LLM reasoning for inappropriate exclusions if any are identified. We recommend that inappropriate exclusions by the LLM triggers a critique of the citation, the LLM prompts and reasoning by the systematic review authors to evaluate whether there is a need to clarify the main screening question prompts or conduct additional fine‐tuning to help the model perform better.

Further evaluation of the GREP‐Agent framework is needed. For the EpiParameter Community we plan to test the GREP‐Agent on other pathogens to be included in the repository for which several systematic reviews have already been undertaken [5, 6, 7, 8]. The application of GREP‐Agent to other topic areas is also needed to further validate the GREP‐Agent performance and adaptability. We invite other researchers to build upon this proposed approach to further refine and optimize performance.

## 5 Conclusions

The integration of AI into evidence synthesis has been studied for some time [21, 22, 23], but with the advent of LLMs there is an opportunity to design processes for using AI to assist in repetitive steps of the review process without training datasets and to strategically include the human reviewer as an active participant. The GREP‐Agent is a novel agentic AI framework that explores and demonstrates improvement in the efficiency and effectiveness of AI‐assisted literature screening in evidence synthesis processes. Future work will look to further the development of best practices for validating, monitoring, and prompting LLMs for evidence synthesis. It is critical that there is investment into development and refinement of adaptable AI systems, such as the GREP‐Agent, to continue to progress the integration of AI into the evidence synthesis process as these tools are invaluable when responding to new or re‐emerging public health threats.

## Author Contributions

**Bing Hu:** conceptualization, investigation, writing – original draft, methodology, validation, visualization, writing – review and editing, software, formal analysis, data curation. **Emmalie Tomini:** conceptualization, writing – original draft, methodology, writing – review and editing, software, data curation. **Tricia Corrin:** conceptualization, data curation, formal analysis, investigation, methodology, writing – original draft, writing – review and editing. **Kusala Pussegoda:** conceptualization, investigation, methodology, data curation, writing – original draft, writing – review and editing. **Elias Sandner:** conceptualization, methodology, writing – review and editing. **Andre Henriques:** writing – review and editing. **Alice Simniceanu:** writing – review and editing. **Luca Fontana:** writing – review and editing. **Andreas Wagner:** writing – review and editing. **Stephanie Brazeau:** conceptualization, funding acquisition, writing – original draft, writing – review and editing, supervision, methodology, project administration, resources. **Lisa Waddell:** conceptualization, data curation, formal analysis, funding acquisition, investigation, methodology, project administration, resources, supervision, writing – original draft, writing – review and editing.

## Conflicts of Interest

The authors declare no conflicts of interest.

## Peer Review

The peer review history for this article is available at https://www.webofscience.com/api/gateway/wos/peer-review/10.1002/cesm.70042.

## Supporting information

Cochrane‐GREP‐EXP‐Screening Appendix.

coi disclosure Waddell‐2025‐02‐28.

CESM+Declaration+of+Interest FORM.

## Data Availability

The data that supports the findings of this study are openly available in The Open Science Framework at https://doi.org/10.17605/OSF.IO/7K4GU.
