# Supplementary material for: Enhancing Evidence Synthesis Efficiency: Leveraging Large Language Models and Agentic Workflows for Optimized Literature Screening
Source: Cochrane Evid Synth Methods. 2025 Oct 21;3(6):e70042. doi: 10.1002/cesm.70042 (PMC12538819; doi:10.1002/cesm.70042)
Supplement: Supplementary file 1 — Cochrane‐GREP‐EXP‐Screening Appendix. [file CESM-3-e70042-s003.pdf]

# Appendix

This is the appendix for the manuscript "Enhancing Evidence Synthesis Efficiency: Leveraging Large Language Models and Agentic Workflows for Optimized Literature Screening" by Hu et al. (2025)

## Contents

A. Agentic Artificial Intelligence ........ 1
B. Measles Dataset ........ 1
C. LLM Selection and Hyperparameters ........ 10
D. System Prompts ........ 11

## A. Agentic Artificial Intelligence

Agentic AI refers to AI systems designed to operate autonomously, and actively plans, executes and adapts based on specific goals. This approach can evaluate multiple options and select the best course of action, it can handle muti-step workflows with minimal human interaction and it continuously refines its approach based on new data or feedback as shown in Figure 2 of the manuscript.

In a multi-agent system, each agent performs a specific subtask required to reach the goal and then their efforts are coordinated through AI automation of tasks to complete without human intervention. In the case of GREP-EXP there is a set of workflow rules that determine disagreements between screening and critical agents, when the ensemble agents are triggered and when citations are passed to human review due to unresolved disagreement and low confidence.

## B. Measles Dataset

GREP-Agent was validated using results from a large measles title and abstract screening dataset from an ongoing systematic review on the epidemiological parameters for measles (PROSPERO CRD42025648371). A clean subset of the title and abstract screening results (2000 citations screened by two independent reviewers) based on pre-defined inclusion and exclusion criteria was used as the human-reviewed control dataset for this project. The smaller dataset was used to provide enough of a variety of citations while balancing the cost and time required to conduct many different model runs.

The title and abstract screening consisted of three questions that would determine if the citation was eligible for inclusion in the systematic review: 1) is this article primary

research? 2) is this article on the human population? and 3) is the main focus of this study about measles disease? The questions along with the possible answers and the guidance provided to the human reviewer to take into consideration when answering the screening questions can all be found in the systematic review protocol.

The dataset was exported from the systematic review software DistillerSR to a CSV file format, a desired citation format which could be inputted into GREP-Agent. The CSV file contained all of the bibliographic information for the citation (e.g., title, abstract, journal, year of publication) in addition to the answers to the title and abstract screening questions. The human reviewer dataset and systematic review protocol can be found here on Open Science Framework: https://doi.org/10.17605/OSF.IO/7K4GU.

### *B.1 Citation Format*

From the 33 available columns provided in the dataset, eight were selected for our citation formatting that were relevant for LLM-screening: title, abstract, keywords, journal, type, type of work, notes, and year. An example can be found below:

**Title**: Viral diseases in South-East Asia and the Western Pacific. Proceedings of an International Seminar on Viral Diseases in South-East Asia and the Western Pacific, Canberra, Australia, 8-12 February 1982
**Abstract**: This is an abridged contents list of the book; papers for which the names of the authors are given are abstracted separately [see the author index for the abstract numbers].
**Keywords**: Parasites, Vectors, Pathogens and Biogenic Diseases of Humans [VV200] hemorrhagic fevers infections parasites parasitosis protozoal infections toxoplasmosis viral diseases arboviruses interferons antiviral agents Protozoa Toxoplasma viruses South East Asia USA Asia Australasia Guatemala Hawaii Oceania Pacific Islands Pacific Ocean eukaryotes Sarcocystidae Eucoccidiorida Apicomplexa APEC countries high income countries North America America OECD Countries very high Human Development Index countries CACM Central America Latin America medium Human Development Index countries upper-middle income countries Pacific States of USA Western States of USA Polynesia arthropod-borne viruses hemorrhagic fevers parasitosis viral diseases in South-East Asia and Western Pacific Western Pacific antivirals parasitic diseases parasitic infestations protozoal diseases viral infections Southeast Asia United States of America Journal: Viral diseases in South East Asia and the Western Pacific. Proceedings of an International Seminar on Viral Diseases in South East Asia and the Western Pacific, Canberra, Australia
**Journal:**
**Type**: JOUR

**Type of Work**: Conference proceedings
**Notes**: Mackenzie, J. S. Viral diseases in South-East Asia and the Western Pacific. Proceedings of an International Seminar on Viral Diseases in South-East Asia and the Western Pacific, Canberra, Australia, 8-12 February 1982. Using Smart Source Parsing 8-12 February .; 1982. xx + 751pp
**Year**: 1982

### *B.2 Measles Screening Criteria*

As stated above, the title and abstract screening was comprised of three criteria questions. Overall inclusion/exclusion can be computed by combining the answers from the three criteria questions.

Individual instances of GREP-Agent were deployed for each criteria question where the LLM must select an answer from the given possible answers for each criteria question. Context for each criteria possible answer is provided to the LLM for additional guidance, nuance, and context. Screening criteria can be subdivided into multiple criteria categories and questions which GREP-Agent can be individually applied to. Along with the desired criteria questions, GREP-Agent requires all possible answers for each question with any additional contextual information to help the LLM to understand when to select that answer.

### *B.3 Initial Measles Screening Criteria*

The initial measles criteria used by the LLM prior to fine-tuning was based off the questions, answers, and guidance provided to the human reviewers in the systematic review protocol for answering the title and abstract screening questions. The following are the initial measles criteria used prior to fine-tuning:

| **Question:** Is this article primary research? | |
|---|---|
| **Possible Answer** | **Context** |
| Yes - primary research | Primary research: a study where the authors collected and analyzed their own data.<br>We will include journal articles, PhD/MSc Theses, reports, and letters to the editor, short communications, and commentaries if they contain primary data.<br>We are excluding conference proceedings/abstracts/posters although they are primary research. *note – we are including models and surveillance reports as primary research.<br>We answer with option \"Yes - primary research\". |

| | |
|---|---|
| No - systematic review meta-analysis or rapid review | We ONLY want to include systematic review-meta analyses and rapid reviews that summarize the evidence on outcomes of interest. If this is any other type of review, please exclude (e.g., scoping, narrative). We answer with option \"No - systematic review meta-analysis or rapid review\".<br>Systematic-review: employ systematic and explicit methods to identify, select, and critically appraise relevant evidence to answer a clearly defined research question.<br>Meta-analysis: a statistical technique that combines the results of individual quantitative studies to provide a precise effect of the result.<br>Rapid review: a type of knowledge synthesis that accelerates the process of conducting a traditional systematic review through streamlining or omitting specific methods to produce synthesis for stakeholders in a timely manner." |
| No - non primary (exclude) | Non-primary: includes letters to the editor, commentaries, opinion pieces, news articles, that DO not contain primary data (report on others work).<br>Also includes narrative literature review and evidence syntheses that would not summarize the outcomes of interest e.g. scoping reviews.<br>We answer with option \"No - non primary (exclude)\". |
| Yes - conference proceeding/abstract/poster (exclude) | For any computer science (i.e. ACM) or math related papers, conference proceedings, conference posters, or conference abstracts we answer with option \"Yes - conference proceeding/abstract/poster (exclude)\"." |

| **Question:** Is this article on the human population? | |
|---|---|
| **Possible Answers** | **Context** |
| Yes | We ONLY want to include studies on the human population. If a review summarizes studies on humans, we answer with option \"Yes - human population\". " |
| No (exclude) | If this is an in vitro study or only on animals, please exclude. To exclude a study we answer with option \"No (exclude)\"." |

| **Question:** Is the main focus of this study about measles disease? | |
|---|---|
| **Possible Answers** | **Context** |

| | |
|---|---|
| Yes | We only want to capture studies where there is a focus is on measles disease, we answer with option \"Yes\".<br>Measles: also known as rubeola, Morbilli, red/English measles is a highly contagious disease that results from infection with the measles virus.<br>Symptoms include fever, cough, runny nose and a rash.<br>Some long-term longitudinal studies can have multiple disease focuses including measles. These studies summarize results collected from multiple diseases over large time periods. We want to include these studies" |
| No (exclude) | Please exclude all studies where the study does not focus on measles disease which includes studies that report on a different disease but mention measles briefly in the abstract (e.g., measles is listed alongside a number of other diseases), studies on knowledge, attitudes, and behaviors on measles/vaccination against measles, studies that only evaluate measles vaccines (e.g., cellular responses of mice immunized with a measles vaccine), and studies reporting chronic conditions of measles (e.g. Subacute sclerosing panencephalitis).<br>Please exclude all studies where the main focus of the study is clearly not about measles disease which includes studies that report on a different disease but mention measles briefly in the abstract (e.g., measles is listed alongside a number of other diseases), studies on knowledge, attitudes, and behaviors on measles/vaccination against measles, studies that only evaluate measles vaccines (e.g., cellular responses of mice immunized with a measles vaccine), and studies reporting chronic conditions of measles (e.g. Subacute sclerosing panencephalitis).<br>To exclude a study we answer with option \"No (exclude)\". |

### *B.4 Fine-Tuned Measles Screening Criteria*

Human feedback for each criteria question collected during the fine-tuning and operational phase of GREP-Agent was used to refine the context for the relevant criteria question and possible answers. To do this, the human review team assessed the conflicts between LLM and human answers to generate additional context and prompts for the LLM to be able to answer the screening questions. Given the granularity of possible human feedback and intervention, GREP-Agent allows for fine-grained control over screening behavior capable of modeling necessary nuance for accurate screening.

The following are the measles criteria after fine-tuning:

| **Question:** Is this article primary research? | |
|---|---|
| **Possible Answer** | **Context** |
| Yes - primary research | Primary research: a study where data is collected and/or analyzed by the authors. We will include journal articles, PhD/MSc Theses, reports, and letters to the editor, short communications, and commentaries if they contain primary data and/or describe the results of a study.<br>Answer this option if the study includes a model, risk assessment or is a surveillance report.<br>Do not answer this option if this is a conference proceedings/ abstracts/ posters although they are primary research.<br>Apply the following additional rules in order of most important to least:<br>- Answer this option if there is surveillance data presented for a geographic region or country.<br>- Answer this option if this is an outbreak report that describes the occurrence of several related cases.<br>- Answer this option if this is a case report or case series, which are a detailed description of the clinical and epidemiological characteristics of one or more human cases of disease.<br>- Do not answer this option if this is a summary of many individual studies. |
| No - systematic review meta-analysis or rapid review | Select this option if this is an evidence synthesis such as systematic review, meta-analysis, rapid review and scoping review that summarize the evidence on outcomes of interest.<br>All evidence syntheses employ systematic and explicit methods to identify, select, and critically appraise relevant evidence to answer a clearly defined research question.<br>Meta-analysis is a statistical technique that combines the quantitative results of individual studies to provide a precise summary effect estimate, and should be preceded by an evidence synthesis.<br>If this is any other type of review that does not describe explicit methods for the identification, selection and appraisal of the studies summarized do not select this option and select No- non primary (exclude).<br>Apply the following additional rules in order of most important to least: |

| | |
|---|---|
| | - Select this option if the label systematic review, rapid review, scoping review or meta-analysis is used to describe the review.<br>- Select this option if the review has a structured methodology that indicates a clear research question, a search methodology, how relevant research was identified and included in the review.<br>- Do not select this option if the citation only indicates an evidence synthesis was used to inform the guideline, recommendation, report or other decision analysis, these are No- non primary (exclude).<br>- Do not select this option if the review does not have reproducible methods, and is only described as a "review", "brief review", "systematic analysis", "narrative review", "review of the current situation", "synthesizing evidence", these are No- non primary (exclude). |
| No - non primary (exclude) | Non-primary: includes letters to the editor, commentaries, opinion pieces, news articles, that DO not contain primary data and are usually a summary or commentary of others primary research.<br>Also includes narrative literature reviews or other summaries of several primary research studies.<br>Apply the following additional rules in order of most important to least:<br>- Answer this option if this is a full conference proceedings that represents the citation for all the studies presented at a conference or meeting.<br>- Answer this option if this is a conference abstract that represents non-primary research.<br>- Answer this option if this is a summary of different methodologies or a review of different methodologies.<br>- Answer this option if this is a guidance document even if the guidance is based on a systematic review.<br>- Answer this option if it only describes a program or process without reporting on the results or an evaluation of the program or process.<br>- Answer this option if the paper only describes recommendations or goals from a consultation or meeting. |
| Yes - conference proceeding/abstract/poster (exclude) | Use this answer for conference proceedings of single studies, conference posters, or conference abstracts.<br>Apply the following additional rules in order of most important to least: |

| | |
|---|---|
| | - Do not answer this option if this is a full conference proceedings that represents the citation for all the studies presented at a conference or meeting, these are No- non primary (exclude). |

| **Question:** Is this article on the human population? | |
|---|---|
| **Possible Answers** | **Context** |
| Yes | Answer with this option if the study is on the human population. If a review summarizes studies on humans, we answer with this option<br>Apply the following additional rules in order of most important to least:<br>- Answer with this option if the study doesn't explicitly state it's about humans, but is about human disease or viruses that cause human disease and indicates clinical spectrum, epidemiology, transmission, outbreak, spread. The abstract also should not explicitly state all the results are from animals or in vitro.<br>- Answer with this option if this is a molecular epidemiology study defined as studies that look at the association between genetics and epidemiological risk factors. |
| No (exclude) | Answer with this option if this is an in vitro study, only on the characteristics of the pathogen, only on animals, or there are no human samples.<br>Apply the following additional rules in order of most important to least:<br>- Answer with this option if the study only presents results of a phylogenetic analysis of the pathogen that is not linked to epidemiological data.<br>- Answer with this option if the study is about in vitro research<br>- Answer with this option if the study is focused on veterinary research, only involving animals or plants. |

| **Question:** Is this study reporting on measles disease? / Is the main focus of this study about measles disease? | |
|---|---|
| **Possible Answers** | **Context** |
| Yes | Answer with this option if the study reports on the measles virus or the disease caused by the measles virus. Measles is also known as rubeola, Morbilli, red/English measles. |

| | |
|---|---|
| | Answer this option if outcomes of measles disease are reported even if outcomes for other diseases are also reported in the study.<br>Apply the following additional rules in order of most important to least:<br>- Answer this option if measures of measles occurrence and mortality are reported.<br>- Answer this option if measles is indicated as one of several vaccine-preventable diseases measured in the study.<br>- Do not answer this option if the only outcomes are related to measles vaccination coverage or adverse reactions or vaccination induced immunity. |
| No (exclude) | Answer this option if measles is only listed as one of several diseases in the abstract or keywords if the study is reporting on knowledge, attitude, and behavior outcomes related to measles or vaccination against measles; if the study is only reported measles vaccine outcomes (e.g., post vaccine titers, vaccine coverage in a population, adverse reactions to measles vaccine, animal models of vaccine immunity, in vitro studies of immune response); studies exploring risk factors for epidemiological outcomes (e.g. mortality or occurrence of chronic disease) where measles is one of many risk factors examined; and studies reporting chronic conditions of measles (e.g. Subacute sclerosing panencephalitis).<br>Apply the following additional rules in order of most important to least:<br>- Do not select this option if measures of measles occurrence and mortality are reported, select Yes.<br>- Select this option if all measles outcomes are related to measles vaccine coverage, measles vaccine antibodies, measles vaccine adverse reactions, measles vaccination for prevention of measles.<br>- Select this option if the study is about measles vaccination quality assurance.<br>- Select this option if the study is about measles diagnostic test accuracy.<br>- Select this option if the study is about the evaluation of a measles related program or policy and no outcomes are about measles disease.<br>- Select this option if the study is about a guideline or recommendation for monitoring or surveillance of measles. |

| | |
|---|---|

## C. LLM Selection and Hyperparameters

We select both proprietary and open-source LLMs for our experimentation. GPT4o is a proprietary model from OpenAI and is selected given its current state-of-the-art performance, albeit higher cost. GPT4o-mini is a smaller proprietary version of GPT4o from OpenAI and is a much more affordable option given a large volume of citations to be screened. Given our available hardware, we test open-source local models Llama3.1 8B parameter from Meta and Phi4 14B parameter from Microsoft; Llama3.1 and Phi4 are both state-of-the-art open-source models that can be loaded and run on commercial grade local hardware and affordable cloud virtual machines. From our model selection, we analyze our GREP-Agent framework on various LLM model sizes as well as between open-source and proprietary model options.

### *C.1 Parameter Selection*

Several parameters can be used to alter the behavior of LLMs in GREP-Agent. The seed is an initial value that ensures the reproducibility of the results; using the same seed will produce the same output every time the model is run. Temperature is a value that regulates the randomness of the predictions: a lower temperature makes the modal increasingly deterministic and conservative, while a higher temperature produces more creative but less coherent text. Nucleus sampling, otherwise known as Top-P, is a sampling technique that selects tokens from the smallest possible set of words whose cumulative probability exceeds a threshold p -  this ensures that the model only considers the most probable words while still allowing for diversity in the output.

| Parameter | Screening Agent | Critical Agent | Ensemble Agents |
|---|---|---|---|
| Model | Selected Model | Selected Model | Rand(GPT4o, GPT4o-mini) |
| Seed | 42 | 42 | RandInt(0,10000) |
| Temperature | 0.2 | 0.2 | RandFloat(0, 0.8) |
| Top-P | 1.0 | 1.0 | RandFloat(0.2, 1.0) |

### *C.2 Addressing Data Leakage*

Data leakage poses a significant risk in literature screening and parameter extraction benchmarks that may be used to demonstrate performance, as LLM tool developers may inadvertently train LLM models on benchmark data, compromising fair evaluation. Private or secret sets of literature review screening and parameter extraction benchmarks are needed for fair comparison of LLM models to combat this possibility of “cheating”.  As our

specific criteria and prompts have not been published prior to our study, it is very unlikely that ChatGPT or any of the LLMs we used had already learned the answers for our specific criteria / prompt that we utilized. However, it is not impossible that the LLMs had seen or knew the answers for similar criteria / prompts for a citation. Potential prior knowledge by the model should not substantially affect our conclusions if it exists and would impart be mediated by the GREP-Agent framework.

### C.3 Insights and Strategy

As each evidence synthesis use case is different, each will require different configurations and hyperparameters. However, the generic framework we describe in this study aims to allow the LLM to answer where it is confident and trigger human review when it is not, while being strategic about when to spend more time (extra model runs) trying to be more decisive. Thus maximizing the operational workload reduction that can be realized while not compromising performance. Achieving optimal balance between performance, workload, and cost is described along with, how varying different hyperparameters can influence that balance. Where more ensemble agents results in a high-cost but better performance, and how using different LLMs can result in different performances that results in more or less workload reduction. For example, the ensemble model includes 3 agents, we did test using more agents to see if it improved decisions made on challenging citations but found that >3 did not improve performance and would result in increased financial and environmental costs.

## D. System Prompts

Each agent can have uniquely specified model and hyperparameter. For our experiments, both screening and critical agent utilize the same specified test model, where the ensemble agent utilizes a randomized model from the set of all models test. For all agents we utilize a temperature of 0.2 and top-p of 0.

The following sections cover the systems prompts used for GREP-Agent.

### *D.1 Screening and Ensemble Agent*

You are a highly critical helpful scientific evaluator completing an academic review. Your job is to screen citations and decide whether to include them or exclude them according to a set criteria.

Answer the question "{question}" for the following citation:

{citations}

The answer has to contain exactly one of the following options:

{possible answers}

Select only one of the above options numbering in your answer in between XML tags <answer></answer>. Your answer must contain one of the above options exactly. For example <answer>0</answer>. Apply and only use the following instructions and criteria to help you select the right answer "{question}".

{context}

Give a probability of confidence between 0 to 1 in between XML tags <confidence></confidence>. For example <confidence>0.52</confidence>. Do not overestimate your confidence.

Explain why another option could be likely and give a confidence level between 0 to 1 in between XML tags <other></other>. For example <other>0.52</other>.

Explain your selected answer and confidence level.

### *D.2 Critical Agent*

You are a highly critical helpful scientific evaluator completing an academic review. Your job is to screen citations and decide whether to include them or exclude them according to a set criteria.

Answer the question "{question}" for the following citation:

{citations}

The answer has to contain exactly one of the following options:

{possible answers}

Select only one of the above options numbering in your answer in between XML tags <answer></answer>. Your answer must contain one of the above

options exactly. For example <answer>0</answer>. Apply and only use the following instructions and criteria to help you answer "{question}".

{context}

If your desired answer is not one of the provided options in <option></option>, answer with the number for "None of the above" in XML tags <answer></answer>.

Give a probability of confidence between 0 to 1 in between XML tags <confidence></confidence>. For example <confidence>0.52</confidence>. Do not overestimate your confidence.

Explain your selected answer and confidence level. Explain why another option could be likely and give a confidence level between 0 to 1 in between XML tags <other></other>. For example <other>0.52</other>.

### *D.3 Example Outputs*

#### D.3.1 Screening Agent

The following is an example response from the screening agent using GPT4o.

<answer>2</answer>

<confidence>0.85</confidence>

<other>0.15</other>

The citation provided is for a full conference proceedings that represents the citation for all the studies presented at a conference or meeting. This is indicated by the title and the abstract, which describe the proceedings of an international seminar and mention that the papers are abstracted separately. According to the criteria, this falls under ""No - non primary (exclude)"" because it is a summary of many individual studies presented at the conference and does not represent a single primary research study.

#### AD3.2 Critical Agent

The following is an example response from the critical agent using GPT4o.

<answer>2</answer>

<confidence>0.9</confidence>

This citation is for conference proceedings, which are explicitly mentioned in the title and type of work. The proceedings represent a collection of papers presented at an international seminar. According to the criteria, conference proceedings, abstracts, and posters should be excluded even if they contain primary research. Therefore, the appropriate answer is ""Yes - conference proceeding/abstract/poster (exclude).""

<other>0.1</other>

Another possible option could be ""No - non primary (exclude)"" because the citation represents the full conference proceedings for all studies presented at the seminar. However, given the specific instruction to use ""Yes - conference proceeding/abstract/poster (exclude)"" for conference proceedings of single studies, the confidence in this alternative is lower.
